# Evaluation of a P300-Based Brain-Machine Interface for a Robotic Hand-Orthosis Control

**DOI:** 10.3389/fnins.2020.589659

**Published:** 2020-11-27

**Authors:** Jonathan Delijorge, Omar Mendoza-Montoya, Jose L. Gordillo, Ricardo Caraza, Hector R. Martinez, Javier M. Antelis

**Affiliations:** ^1^Tecnologico de Monterrey, Escuela de Ingeniería y Ciencias, Monterrey, Mexico; ^2^Tecnologico de Monterrey, Escuela de Medicina y Ciencias de la Salud, Monterrey, Mexico

**Keywords:** brain-machine interface, electroencephalography, evoked potentials, P300, amyotrophic lateral sclerosis, signal processing, artificial intelligence, hand-orthosis

## Abstract

This work presents the design, implementation, and evaluation of a P300-based brain-machine interface (BMI) developed to control a robotic hand-orthosis. The purpose of this system is to assist patients with amyotrophic lateral sclerosis (ALS) who cannot open and close their hands by themselves. The user of this interface can select one of six targets, which represent the flexion-extension of one finger independently or the movement of the five fingers simultaneously. We tested offline and online our BMI on eighteen healthy subjects (HS) and eight ALS patients. In the offline test, we used the calibration data of each participant recorded in the experimental sessions to estimate the accuracy of the BMI to classify correctly single epochs as target or non-target trials. On average, the system accuracy was 78.7% for target epochs and 85.7% for non-target trials. Additionally, we observed significant P300 responses in the calibration recordings of all the participants, including the ALS patients. For the BMI online test, each subject performed from 6 to 36 attempts of target selections using the interface. In this case, around 46% of the participants obtained 100% of accuracy, and the average online accuracy was 89.83%. The maximum information transfer rate (ITR) observed in the experiments was 52.83 bit/min, whereas that the average ITR was 18.13 bit/min. The contributions of this work are the following. First, we report the development and evaluation of a mind-controlled robotic hand-orthosis for patients with ALS. To our knowledge, this BMI is one of the first P300-based assistive robotic devices with multiple targets evaluated on people with ALS. Second, we provide a database with calibration data and online EEG recordings obtained in the evaluation of our BMI. This data is useful to develop and compare other BMI systems and test the processing pipelines of similar applications.

## 1. Introduction

Since the early developments of BMIs, one of the most promising applications of this technology is the use of neuroprosthetic devices to assist people with reduced mobility. There is a consensus among researchers of this area that BMIs may significantly improve the lives of patients who suffer neuromuscular disorders such as ALS. Even so, despite all the efforts in the last three decades to design and implement reliable BMI systems, the goal of developing functional neuroprostheses has not been reached yet. Researchers and engineers must solve many technical and practical problems before bringing this technology into everyday life. Some open issues concerning the development of robust brain-controlled applications are the ability of the system to interpret the user's intentions accurately, the time to process and analyze brain signals, and the stability of performance over time (Murphy et al., 2016).

A BMI is a system that translates cerebral activity into commands to communicate with an external device, bypassing the normal neuromuscular pathways (Wolpaw et al., 2002; Aydin et al., 2018). There are various techniques to register brain signals, but the non-invasive neuroimage modality most widely used in BMI applications is electroencephalography (EEG) because of its high temporal resolution, low cost, and mobility (Flores et al., 2018; Xiao et al., 2019). Among EEG-based BMIs, the P300 paradigm is one of the most popular techniques for building applications with multiple options because it allows achieving high accuracies without the need for long calibration sessions (Hwang et al., 2013; De Venuto et al., 2018). Compared with other paradigms, P300-based BMIs have higher bit rates than motor imagery interfaces, while the stimulation technique for evoking P300 potentials is less visually fatiguing than the method used to elicit steady-state visually evoked potentials (Cattan et al., 2019).

The P300 signal is an event-related potential (ERP) component observed in the electroencephalogram elicited about 300 ms after the perception of an oddball or relevant auditory, visual, or somatosensory stimulus (Cattan et al., 2019). Typically, in a P300-based BMI, characters, syllables, or icons presented on a computer screen flash randomly one at a time while the user focuses attention on one particular graphical element (target stimulus). Each flashing stimulus represents an option, action, or command that the system can execute. The user selects one option of the interface by counting or performing a cognitive task every time the target stimulus is highlighted. Because the target option flashes randomly, this stimulus produces a P300 evoked potential synchronized with the flickering event in the timeline. In this way, a P300-based BMI identifies which option is evoking an ERP to decode the user's intentions and perform the desired action.

Numerous published works have reported examples of P300-based BMIs for communication and control, including spellers (Kleih et al., 2016; Poletti et al., 2016; Okahara et al., 2017; Flores et al., 2018; Guy et al., 2018; Deligani et al., 2019; Shahriari et al., 2019), authentication systems (Yu et al., 2016; Gondesen et al., 2019), assistive robots (Arrichiello et al., 2017), smart home environments (Achanccaray et al., 2017; Masud et al., 2017; Aydin et al., 2018), neurogames (Venuto et al., 2016), remote vehicles (De Venuto et al., 2017; Nurseitov et al., 2017), wheelchairs (De Venuto et al., 2018), and robotic arms (Tang et al., 2017; Garakani et al., 2019). Because the development of assistive technologies for motor-impaired people is one of the major purposes of BMI research, some groups have evaluated similar applications in clinical environments on people with neurological disorders or reduced mobility. Regarding medical applications, we can find P300-based BMIs for ALS (Liberati et al., 2015; Schettini et al., 2015; Poletti et al., 2016; Guy et al., 2018; Deligani et al., 2019; Shahriari et al., 2019; McFarland, 2020), Alzheimer's (Venuto et al., 2016), spinocerebellar ataxia (Okahara et al., 2017), and post-stroke paralysis (Kleih et al., 2016; Achanccaray et al., 2017; Flores et al., 2018). Recently, P300-based BMIs have also been proposed for rehabilitation contexts (Kleih et al., 2016), and diagnosis/evaluation purposes (Poletti et al., 2016; Venuto et al., 2016; Deligani et al., 2019; Shahriari et al., 2019).

Some studies have stated the benefits of orthoses for ALS patients (Tanaka et al., 2013; Ivy et al., 2014); however, the implementation of BMI-controlled robotic hand-orthoses for this target population remains underexplored in comparison to the application of these systems for other neuromotor disorders. Moreover, most of the recent published BMIs for ALS are designed for communication purposes (Vaughan, 2020). Similarly, while the employment of BMI-controlled hand-orthoses is well-known in other neuromotor conditions (e.g., stroke recovery), the effect of the use of these systems in ALS patients remains poorly investigated. A critical step toward the development of practical robotic neuroprostheses for people with ALS is the evaluation of this technology in different scenarios. It is essential to determine if ALS patients can operate this particular mind-controlled application and evaluate the possible effect of a hand-orthosis on the user's experience and performance.

This work presents the development and evaluation of a P300-based BMI coupled with a robotic hand-orthosis device. The purpose of this system is to assist people with ALS to perform movements of individual fingers of one hand, or more complex tasks that involve a sequence of actions of one or more fingers. Eighteen healthy participants and eight ALS patients conducted an experiment in which they tested the proposed BMI selecting a sequence of actions that the robotic hand-orthosis executed. In the evaluation of this BMI, we considered six types of operations: the flexion-extension of individual fingers, and the flexion-extension of the five fingers simultaneously.

In the experiments, we recorded the data used in the training phase of the BMI, and the EEG signals measured during the online tests. The training data was used to evaluate offline the performance of the classification model implemented in the BMI to discriminate between target and non-target epochs. Additionally, we analyzed the P300 responses of the participants to determine if there are subjects without clear evoked potentials. In the online tests, we calculated the classification accuracy and the selection times of the BMI. It is important to say that some selections were made without connecting the hand-orthosis to the system to evaluate the effect of the robotic device in the online accuracy of the BMI.

To our knowledge, our system is the first P300-based BMI that allows ALS patients to perform sequences of movements of individual or two or more digits simultaneously; it is important to consider the advantage of our system to allow the individual movement of the digits since ALS is associated with the degeneration of the corticospinal tract (Sarica et al., 2017) that allows to perform the fine finger motor tasks (Levine et al., 2012). Besides, being a P300-based system, the calibration precises a minimum time consuming calibration, reducing the fatigue of patients in comparison with other systems.

Another contribution of this work is the dataset obtained in the experimental sessions of the proposed BMI. This dataset contains the training data and the online recordings of 26 participants. The calibration samples are useful to evaluate different machine learning models of P300-based BMIs, whereas the online signals can be used to test practical systems without the need for real participants. The relevance of this database resides in the importance of providing high-quality EEG observations that represent both control and ALS groups. Any researcher may evaluate other P300-based BMIs and verify if their proposals can correctly identify the user's intentions in online conditions.

The remainder of this paper is divided into three sections. Section 2 describes the hardware and software components of the mind-controlled hand-orthosis, and the experimental setup under which we tested the BMI. Section 3 shows the results obtained from the system evaluation, while section 4 discusses the implications of the results and the conclusions derived from this work.

## 2. Materials and Methods

### 2.1. Brain-Machine Interface

The proposed system consists of a P300-based BMI coupled with a Hand Of Hope robotic arm (Rehab-Robotics Company, China). This hand-orthosis is a therapeutic device with five DC linear motors designed initially for the rehabilitation of post-stroke patients (Aggogeri et al., 2019). There is a detailed description of the Hand of Hope and its functionality in Ho et al. (2011). To communicate the orthosis with the BMI, we enabled a wireless communication channel to send the position of each motor during the execution of one movement or sequence of movements. In this way, the user selects one action to perform with the hand-orthosis using the P300-based interface.

**Figure 1:**
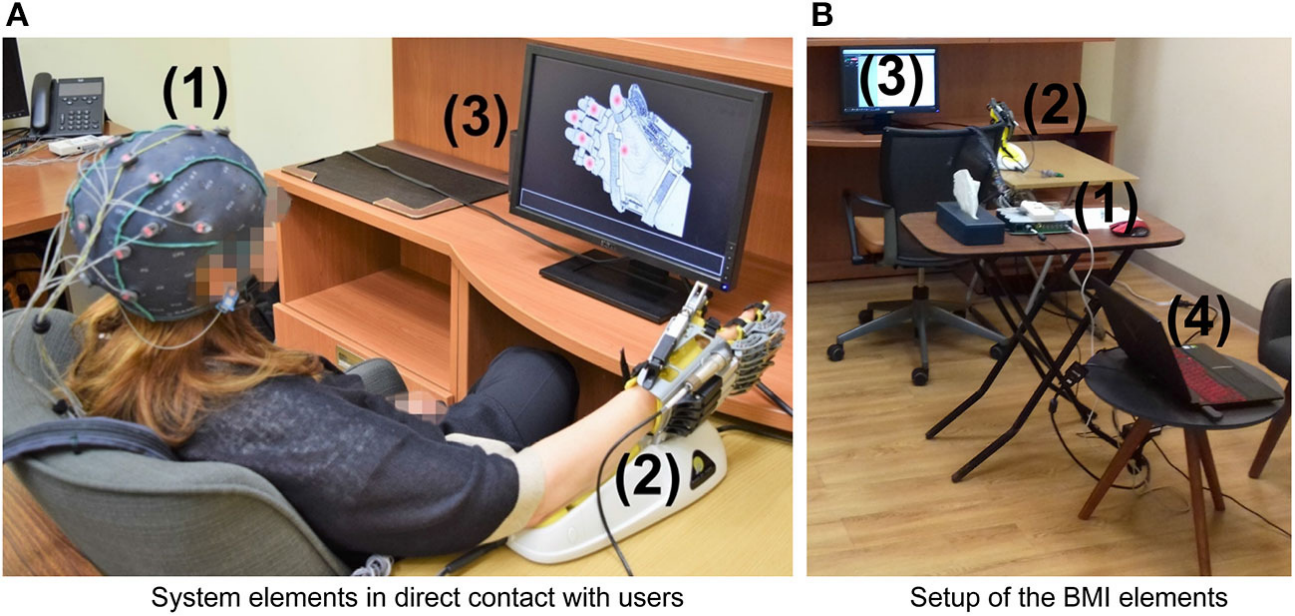
Hardware components of the BMI: (1) EEG recording system (EEG cap, active electrodes, and amplifier), (2) Hand of Hope orthosis, (3) monitor to display the GUI, and (4) computer to process EEG signals, synchronize the stimuli, and send control commands. **(A)** System elements in direct contact with users. **(B)** Setup of the BMI elements.

Figure 1 sketches the components of the mind-controlled hand-orthosis, and how the users interact with them. The main hardware components of the interface are:

- An EEG recording system (a g.GAMMASYS active wet electrode arrangement and a g.USBamp amplifier provided by g.tec medical engineering GmbH, Austria). For this study, the sampling rate was 256 Hz, and we used eight monopolar electrodes, placed according to the 10–20 international system at positions Fz, Cz, P3, Pz, P4, PO7, PO8, and Oz. The ground electrode was located at AFz, and the reference electrode on the right earlobe.
- A Hand of Hope robotic arm. The users can wear the robotic device on any hand.
- A monitor that displays the graphical user interface (GUI) of the BMI.
- A computer that processes the EEG signals, synchronizes the stimulus presentation, and sends the control commands to the hand-orthosis.

The software elements of this system, including the GUI, were implemented in-house using C++.

**Figure 2:**
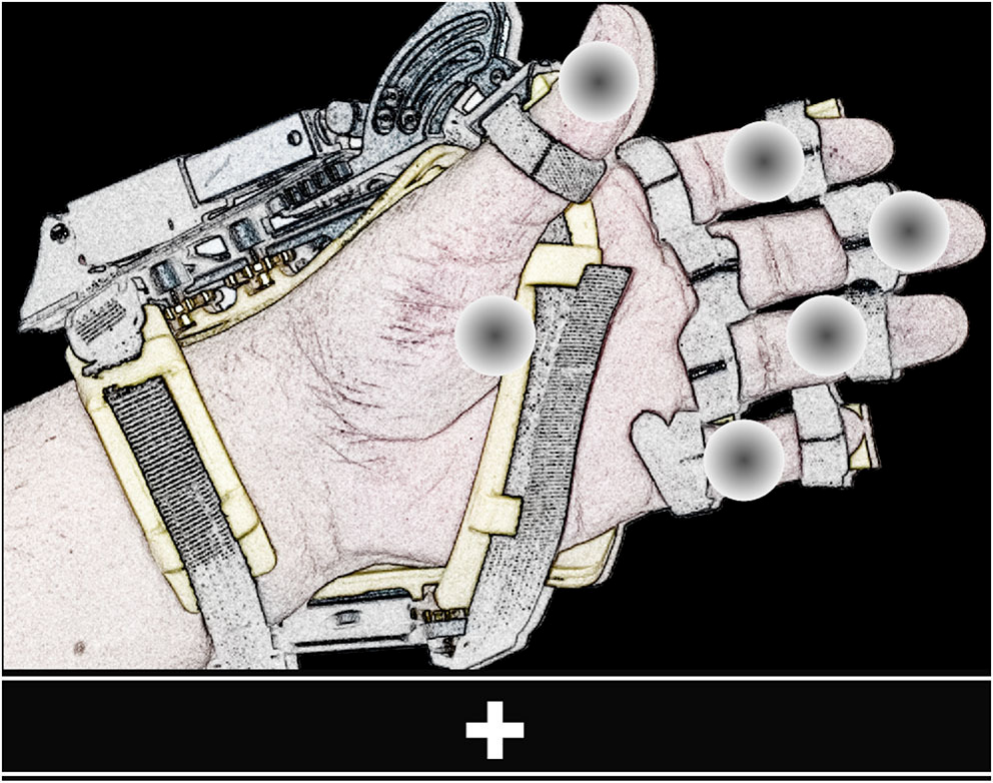
View of the GUI. The screen shows six flashing gray circles (possible options) placed over the image of a left or right hand wearing the orthosis. The bar located at the lower part of the GUI presents the instructions to operate the BMI and feedback.

The GUI of the BMI (shown in Figure 2) provides the instructions to operate the system, presents the flashing elements, and displays visual feedback. In this GUI, gray circles positioned on a graphical illustration of the hand-orthosis represent the available options (i.e., actions or movements of the robotic device). Since the orthosis can be used on any hand, the GUI can display the image of a left or right hand, according to the side where the robotic device would be placed.

It is possible to program different movements or actions for the hand-orthosis. The system can move each finger independently or perform multiple movements at the same time. For this study, we evaluated the BMI using six options: the individual flexion-extension of each finger, and the simultaneous flexion-extension of the five fingers. The five gray circles placed over the fingers represent the individual movements, whereas the circle over the palm corresponds to the hand opening and closing.

**Figure 3:**
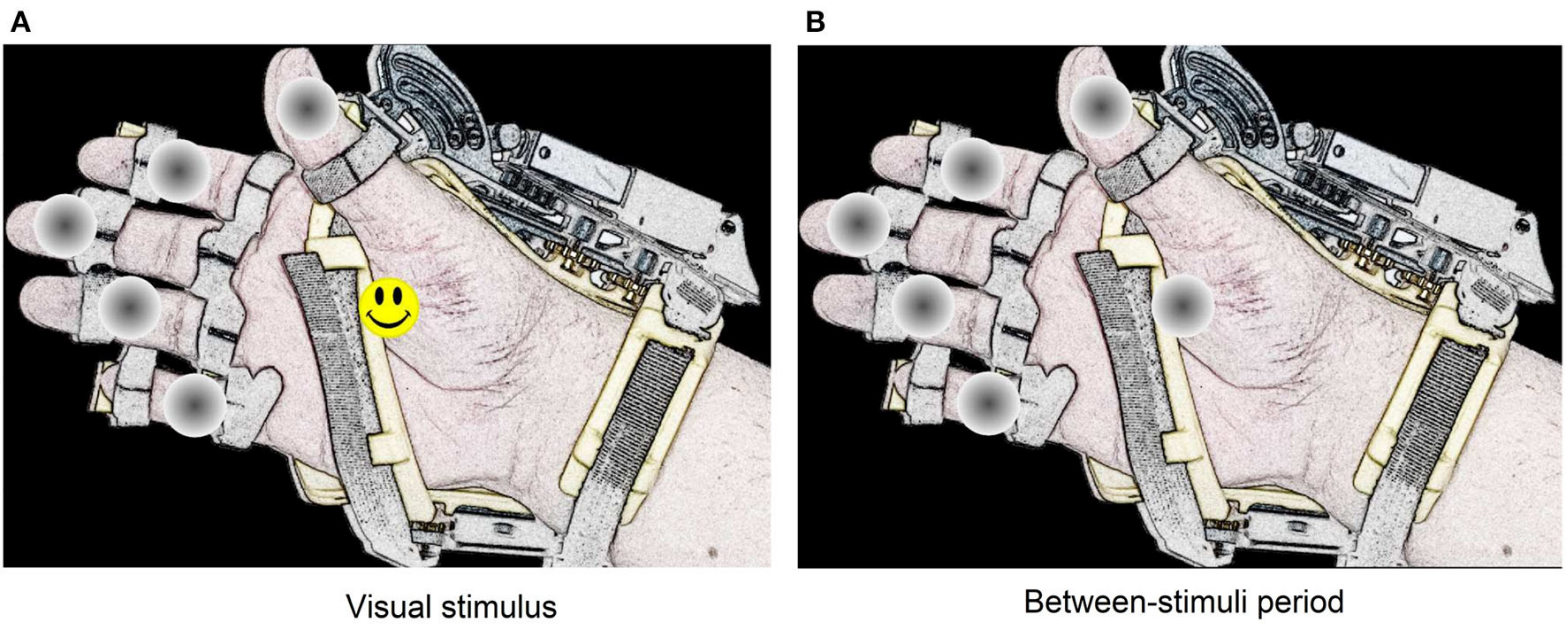
Representation of the dummy face pattern method for visual stimulation. The visual stimulus remain active during 75 ms on one option selected randomly **(A)**. Between each stimulus, there is a period of 75 ms where all circles remain gray colored **(B)**.

In this system, the stimulation method used to elicit evoked responses is the dummy face pattern (Chen et al., 2015, 2016), which consists of a yellow happy face icon that replaces for a short time a gray circle selected randomly. In one flashing cycle, the happy face icon is shown for 75 ms, and then all the gray circles appear for 75 ms (see Figure 3). The users are instructed to choose freely one movement or action of the robotic device by counting how many times the happy face is displayed on the desired option. If the system detects a P300 response for one action, the flashing stops for 4 s, while the hand-orthosis performs the corresponding movement. Then, the interface restarts the random flashing and waits for another evoked response. The same routine is repeated continuously during the regular operation of the BMI.

**Figure 4:**
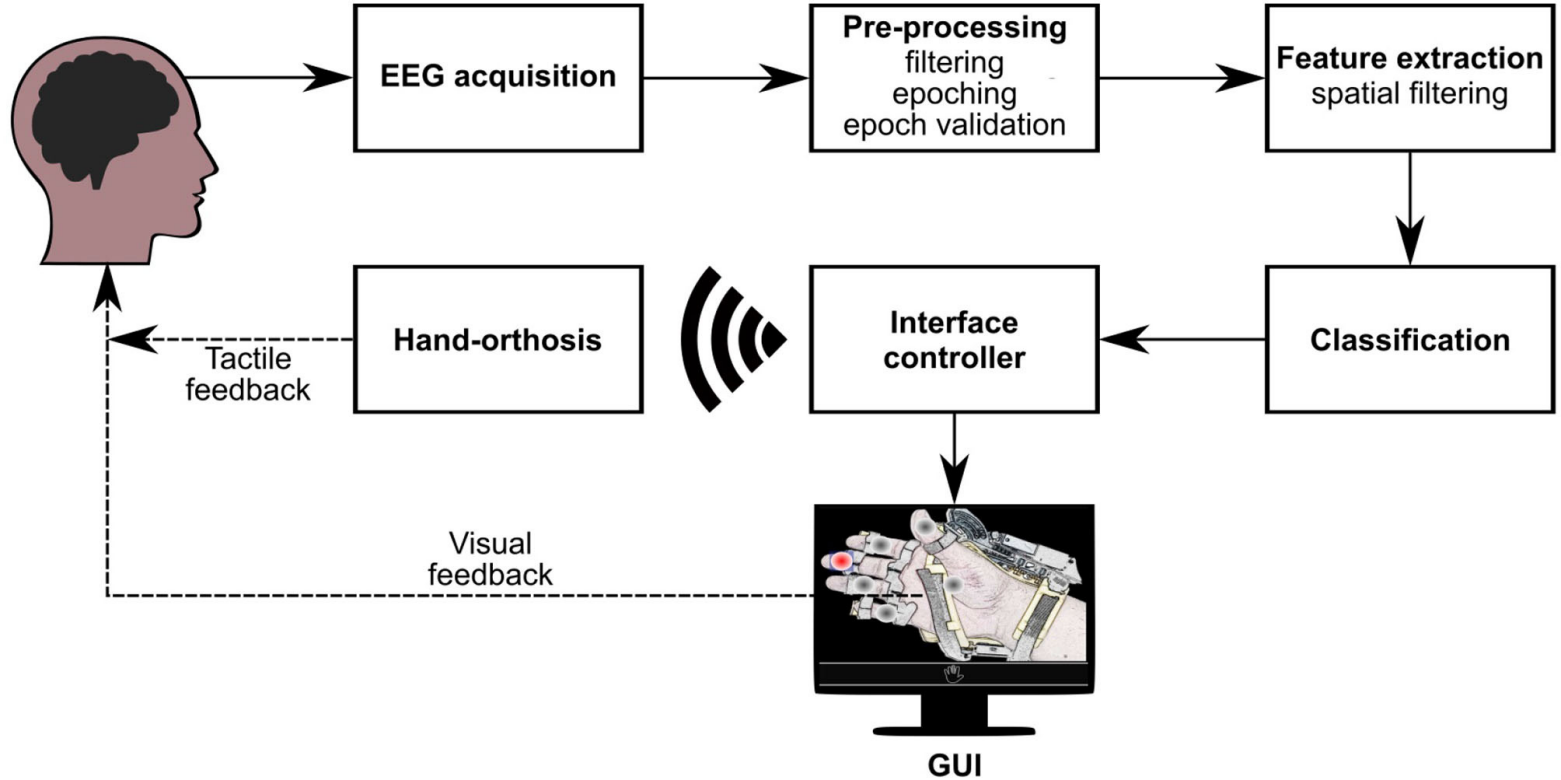
Processing stages of the BMI. Once acquired the EEG signals, they go through the pre-processing, feature extraction, and classification stages. Based on the results of the classifier, the interface controller synchronizes the visual stimuli and sends control commands via WiFi to the orthosis. If the BMI detects a target option, the GUI provides visual feedback to the user, and the orthosis provides tactile feedback.

The detection of evoked responses consists of a sequence of processing steps necessary to extract relevant information from the measured EEG signals. Figure 4 summarizes the different stages implemented in our BMI to analyze and classify electrophysiological data. Firstly, when one option flashes, the system extracts the EEG epoch (or trial) around this event and applies some pre-processing and feature extraction techniques on this data segment. Then, a classification model evaluates the computed characteristics to obtain the label that represents the class of the processed epoch (target or non-target). A third class is also considered in this design (artifact) to indicate if a trial is contaminated by noise or muscle artifacts. Finally, the BMI processes this label to determine whether the flashing stimulus is eliciting evoked responses. If there is a P300 evoked potential, the BMI sends the respective control signals to the robotic device. This processing pipeline is based on the classification approach described in Mendoza-Montoya (2017).

The following describes the processing stages of the BMI, and the component of the interface that interacts with the robotic device. Also, we present the calibration routine implemented to train the system.

#### 2.1.1. EEG Pre-processing

In the pre-processing stage, the BMI extracts three band-limited components using FIR filters with cut-off frequencies between 4 and 14, 20–40, and 4–40 Hz. Then, when one flashing occurs, the samples around the time-window of the event are separated so that the epoched signals contain 800 ms of post-stimulus samples, starting from the stimulus onset. The result of this processing step are signals $X^{4-14}=\left[x_{e}^{4-14}\left(t\right)\right]\in {\mathbb{R}}^{n_{e}\times n_{t}}$, $X^{20-40}=\left[x_{e}^{20-40}\left(t\right)\right]\in {\mathbb{R}}^{n_{e}\times n_{t}}$, and $X^{4-40}=\left[x_{e}^{4-40}\left(t\right)\right]\in {\mathbb{R}}^{n_{e}\times n_{t}}$, where *e* represents the electrode position (*e* = 1, 2, 3, …, *n*_*e*_), *n*_*e*_ is the number of electrodes or channels, *t* is the time index (*t* = 1, 2, 3, …, *n*_*t*_), and *n*_*t*_ is the number of samples.

The next step is the epoch validation, which is necessary to determine if the EEG trial is not contaminated by muscle artifacts or other sources of noise. Here, the BMI calculates the peak-to-peak voltage $v_{e}^{pp}$, the standard deviation σ_*e*_, and the power ratio *r*_*e*_ of each channel as follows:

(1) $$\begin{array}{l} v_{e}^{pp}={\text{max}}_{t}\left(x_{e}^{4-40}\left(t\right)\right)-{\text{min}}_{t}\left(x_{e}^{4-40}\left(t\right)\right), \end{array}$$

(2) $$\begin{array}{l} \sigma_{e}=\sqrt{\frac{1}{n_{t}-1}\sum_{t=1}^{n_{t}}{\left(x_{e}^{4-40}\left(t\right)-\mu_{e}\right)}^{2}}, \end{array}$$

(3) $$\begin{array}{l} r_{e}=\frac{\sum_{t=1}^{n_{t}}{\left(x_{e}^{20-40}\left(t\right)\right)}^{2}}{\sum_{t=1}^{n_{t}}{\left(x_{e}^{4-40}\left(t\right)\right)}^{2}}, \end{array}$$

where

(4) $$\begin{array}{l} \mu_{e}=\frac{1}{n_{t}}\overset{n_{t}}{\underset{t=1}{\sum }}x_{e}^{4-40}\left(t\right). \end{array}$$

The system classifies as “artifact” any epoch with one or more channels for which $v_{e}^{pp}⩾200$ μV, σ_*e*_ ⩾ 50 μV, or *r*_*e*_ ⩾ 0.7. In this case, the trial is not processed and evaluated by the machine learning model of the BMI. On the other hand, if the epoch passes the validation, i.e., the calculated metrics for all channels are below the threshold levels, the system downsamples *X*^4−14^ using a decimation factor of four to obtain signal $Y=\left[y_{e}\left(t\right)\right]\in {\mathbb{R}}^{n_{e}\times {\hat{n}}_{t}}$, where ${\hat{n}}_{t}$ is the number of time points after the downsampling.

#### 2.1.2. Feature Extraction

The system implements an algorithm of spatial filtering based on canonical correlation analysis (CCA) for feature extraction. This approach is effective in reducing the data dimensionality and increasing the classification accuracy (Spüler et al., 2014; Mendoza-Montoya, 2017). Spatial filtering is a technique that finds linear combinations or projections of a set of signals in such a way that the new signals in the projected space have better separability between classes or another improved property. Given column vector $w=\left[w_{e}\right]\in {\mathbb{R}}^{n_{e}}$ with *n*_*e*_ spatial weights, the projected signal $\tilde{y}$(*t*) is obtained as follows:

(5) $$\begin{array}{l} \tilde{y}\left(t\right)=\overset{n_{e}}{\underset{e=1}{\sum }}w_{e}y_{e}\left(t\right). \end{array}$$

In our BMI, the spatial weights increases the correlation between epochs of the target option and the expected ERP response of this class. Let $Y^{\text{target}}=\left\{Y_{1},Y_{2},Y_{3},\ldots ,Y_{n_{\text{target}}}\right\}$ be a set with *n*_target_ pre-processed observations free of artifacts of the target class obtained from raw calibration data ($Y_{k}=\left[y_{e,k}\left(t\right)\right]\in {\mathbb{R}}^{n_{e}\times {\hat{n}}_{t}}$). The average ERP waveform ${\bar{Y}}^{\text{target}}\in {\mathbb{R}}^{n_{e}\times {\hat{n}}_{t}}$ of these observations is:

(6) $$\begin{array}{l} {\bar{y}}_{e}^{\text{target}}\left(t\right)=\frac{1}{n_{\text{target}}}\overset{n_{\text{target}}}{\underset{k=1}{\sum }}y_{e,k}\left(t\right). \end{array}$$

The training epochs of the target class and their average ERP waveform are concatenated to build matrices $U={\left[Y_{1},Y_{2},\ldots ,Y_{n_{target}}\right]}^{T}$ and $V={\left[{\bar{Y}}^{\text{target}},{\bar{Y}}^{\text{target}},\ldots ,{\bar{Y}}^{\text{target}}\right]}^{T}$ of dimensions $\left({\hat{n}}_{t}\cdot n_{\text{target}}\right)\times n_{e}$, where *T* denotes transpose. CCA is applied to calculate vectors *w* and $\tilde{w}$ that maximize the correlation between *Uw* and $V\tilde{w}$. Here, the system uses *w* as spatial filter to transform the pre-processed epoch.

Because CCA produces *n*_*e*_ spatial filters, the system selects the best *n*_*w*_ projections which correspond to the highest correlation values (1 ⩽ *n*_*w*_ ⩽ *n*_*e*_). To evaluate the system performance, we set *n*_*w*_ = 4. In this way, after the spatial filtering, the BMI obtains the projected signal $\tilde{Y}\in {\mathbb{R}}^{n_{w}\times {\hat{n}}_{t}}$.

#### 2.1.3. Classification

To classify one flashing event, the machine learning model of the BMI evaluates the corresponding signal $\tilde{Y}$ and returns a label or category *L* ∈ {target, non-target}, indicating whether the flickering option is a target stimulus. This operation is only applied to trials free of noise or artifacts. For non-valid epochs, the assigned label is “artifact.”

The system uses the regularized version of the linear discriminant analysis (RLDA) (Lotte and Guan, 2009) to distinguish between the target and non-target epochs. This binary model has been employed previously to detect P300 potentials (Zhumadilova et al., 2017) and classify other electrophysiological responses (Cho et al., 2018). In this stage, the classifier evaluates only a small subset *Z* = {*z*_1_, *z*_2_, …, *z*_*n*_*f*__} of *n*_*f*_ features, selected from the $n_{w}\times {\hat{n}}_{t}$ spatially filtered variables ($z_{i}\in \left\{{\tilde{y}}_{e}\left(t\right)\right\}$). This dimensionality reduction is necessary to prevent over-fitting and reduce the complexity of the machine learning model (Tyagi and Nehra, 2017).

During the system calibration, the BMI chooses the characteristics to evaluate in the classification stage using the forward-backward stepwise (SW) method for variable selection (James et al., 2015). This algorithm starts with an empty classification model without variables and incorporates the one that contributes best to the model performance according to a scoring criterion. Then, the best feature that is not in the model and improves the performance criterion significantly is incorporated. If none of the candidate variables help to enhance the classifier, the model is not modified. In the next step, the variable that is in the model that may be excluded without reducing the actual scoring significantly is removed. Again, the feature set is not altered when it is not possible to discard one feature without worsening the model. These steps are repeated until no more changes in the feature set are possible. In this framework, the features selection and the model training are performed simultaneously.

#### 2.1.4. Interface Controller

The label obtained in the classification stage might be used directly to determine the action or movement to produce with the robotic device. However, because the accuracy of the machine learning model is typically below 90%, the risk of executing an incorrect action is high. It is essential to consider that there is only one target stimulus and multiple non-target options so that before evaluating an epoch of the desired option, the model must detect correctly multiple instances of the non-target class. For this reason, the system processes the history of labels to determine if there is enough evidence that the user wants to select one particular option.

The interface controller is the element of the BMI that receives the labels of the flashing events and determines which action must perform the hand-orthosis. Additionally, it generates the control signals necessary to perform the selected movements or actions and synchronizes the state of the GUI to produce visual feedback. This component decides when the hand-orthosis must be activated and which movement or sequence of movements it must execute.

When the system processes one flashing event, the interface controller evaluates the number of times that each option has been classified as target and non-target responses. Only the last ten flashing events of each flashing symbol free of artifacts are considered in this counting. One option is selected if the following conditions are satisfied:

- The corresponding gray circle of the analyzed option has flashed at least five times (minimum number of processed epochs).
- At least 70% of the flashing events of this option has been classified as target stimuli (target class threshold).
- The responses of each of the other flashing circles have been classified 60% of the time as non-target (non-target class threshold).

If the controller detects a P300 response for one particular option, the flashing sequence is interrupted, providing visual feedback to the user about the selection. Subsequently, the hand-orthosis executes the chosen routine, and the flashing sequence restarts for another selection.

#### 2.1.5. Calibration Routine

The operation of the BMI requires a set of spatial filters and a classification model to process and evaluate the epochs of the flashing events. To find these components, the system provides a calibration routine in which the user focuses attention on target options while the BMI records the subject's brain signals. This routine replicates the operational conditions of the BMI without activating the hand-orthosis. It uses the same GUI with six options, the stimulation method is the dummy face pattern, and the happy face icon appears for 75 ms alternating with 75 ms of no visual stimulus. Because the hand-orthosis is not necessary to train the interface, this device is disabled, and the user is not instructed to wear it.

**Figure 5:**
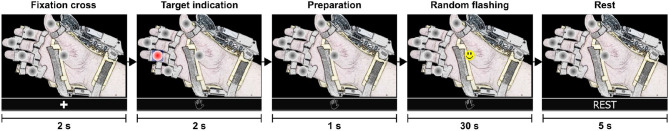
Steps of a single training sequence (training run). The complete calibration consists of eight runs and lasts 320 s. At the end of the calibration routine, the training dataset contains 264 epochs of the target class and 1,320 trials of the non-target class.

The calibration routine is divided into eight training sequences or runs. A run (shown in Figure 5) starts with a fixation cross to indicate that a training sequence has begun. Then, the interface presents a target option (selected randomly by the interface), followed by short preparation time. Next, the options flash randomly one after another for 30 s. Here, the user must count mentally how many times the happy smile icon appears on the specified target option. Finally, the user rests for a few seconds before the next run. At the end, the training dataset contains 264 epochs of the target class and 1,320 trials of the non-target class.

**Figure 6:**
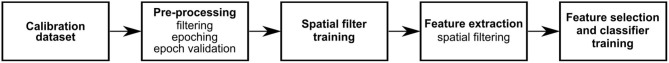
Processing stages for system calibration. The information contained in a calibration dataset is pre-processed and analyzed to obtain a set of spatial filters and a classification model. These two components are necessary to operate the BMI and control the hand-orthosis.

After completing the calibration routine, the system processes and validates the dataset to train the machine learning model of the BMI (see Figure 6). Firstly, the system pre-processes the complete dataset to obtain downsampled epochs free of artifacts of both classes. Next, the spatial filters are calculated using the set of observations of the target class. Then, the calculated filters are applied to the extracted epochs of both classes. Finally, the spatially filtered observations are used to find the optimal subset of features of the classifier and the parameters of this model. Once the classification model is trained, the BMI is ready to operate online and send control commands to the hand-orthosis.

### 2.2. Participants

To evaluate the proposed mind-controlled hand-orthosis, we conducted an experiment in which HS and people with ALS tested the brain-machine interface. In this study, we included eighteen healthy participants (10 females and eight males, aged between 19 and 63 years old, mean age 32.7) and eight ALS patients (three females and five males, aged between 49 and 72 years old, mean age 59.6), all with normal or corrected vision. Table 1 shows the age range of each participant, and the characteristics of the ALS patients. Study subjects had no previous experience with any brain-machine interface.

**Table 1:** Characteristics of the participants.

			**Target detection attempts**					
**Subject**	**Age range** **(years)**	**Without** **orthosis**	**With** **orthosis**	**Total**				
HS1	21-25	12	18	30				
HS2	61-65	18	18	36				
HS3	21-25	12	18	30				
HS4	16-20	12	12	24				
HS5	16-20	12	12	24				
HS6	51-55	18	18	36				
HS7	46-50	12	12	24				
HS8	51-55	18	18	36				
HS9	26-30	18	12	30				
HS10	26-30	12	12	24				
HS11	21-25	18	18	36				
HS12	61-65	18	18	36				
HS13	16-20	18	6	24				
HS14	16-20	18	18	36				
HS15	21-25	18	18	36				
HS16	26-30	18	12	30				
HS17	21-25	18	18	36				
HS18	21-25	18	6	24				
						**ALSFRS-R**	**Years from** **symptoms** **onset**	**Hand** **motor** **impairment**
ALS1	46-50	0	12	12		44	2	mild
ALS2	56-60	0	12	12		35	2	moderate
ALS3	61-65	0	6	6		40	2	moderate
ALS4	56-60	0	12	12		33	2	advanced
ALS5	46-50	18	12	30		26	2	advanced
ALS6	71-75	18	18	36		35	2	moderate
ALS7	61-65	18	18	36		42	2	moderate
ALS8	61-65	12	12	24		39	3	moderate

*This table specifies the age range of each participant and the number of online validation runs performed for each condition of orthosis usage. The column “Total” indicates the total number of validation runs performed among both conditions. For patients, the last three columns indicate the ALS Functional Rating Scale Score (ALSFRS-R) score, the years from the onset of the ALS symptoms, and the level of motor impairment of the hands measured as mild (no observable to sporadic symptoms), moderate (visible symptoms), or advanced (no residual movement)*.

ALS participants were selected from the patients attending the TecSalud ALS Multidisciplinary Clinic (Martínez et al., 2020) considering the disease duration and disability level as inclusion criteria. According to this criterion, the eight participants had, at the time of the tests, a disease duration from 2 to 3 years, and a general disability level ranged from mild to moderate (according to the ALSFRS-R scores). Both, HS and ALS groups volunteered for the study and provided informed consent before the experimental sessions. This study followed the ethical principles of the World Medical Association (WMA) Declaration of Helsinki (WMA, 2013).

### 2.3. Experimental Design

The experiments were carried out in a dedicated medical room at Zambrano-Hellion Medical Center. HS and patients who could walk without help or a wheelchair were asked to sit in a comfortable chair approximately one meter apart from the 22 inch LCD monitor of the BMI. For patients that needed assistance, the room space was adapted to accommodate a wheelchair close to the robotic device in front of the monitor. Before starting the experiments, participants were informed about the general instructions of the different tasks and were asked to avoid unnecessary movements when they had to focus attention on the interface.

**Figure 7:**
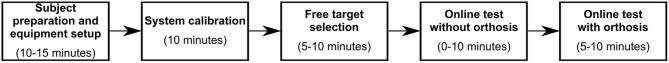
Different stages of the experiment designed for evaluating the mind-controlled hand-orthosis. An experimental session started with the subject preparation and system setup. Then, the participant trained the BMI and tested the interface freely. Finally, the experiment concluded with the online tests. A complete experiment lasted approximately from 30 to 55 min.

Figure 7 summarizes the different stages of one experimental session. After placing the EEG cap and preparing the wet electrodes, the experimenter instructed the participants to calibrate the BMI and perform a short free validation. In this test, subjects selected freely any option of the interface and notified if the system detected the desired action correctly. The purpose of the free selections was to obtain information about the detection times and demonstrate the users that the BMI is effectively responding to their intentions. Participants repeated at least three times the free target selection before continuing with the experiment.

**Figure 8:**
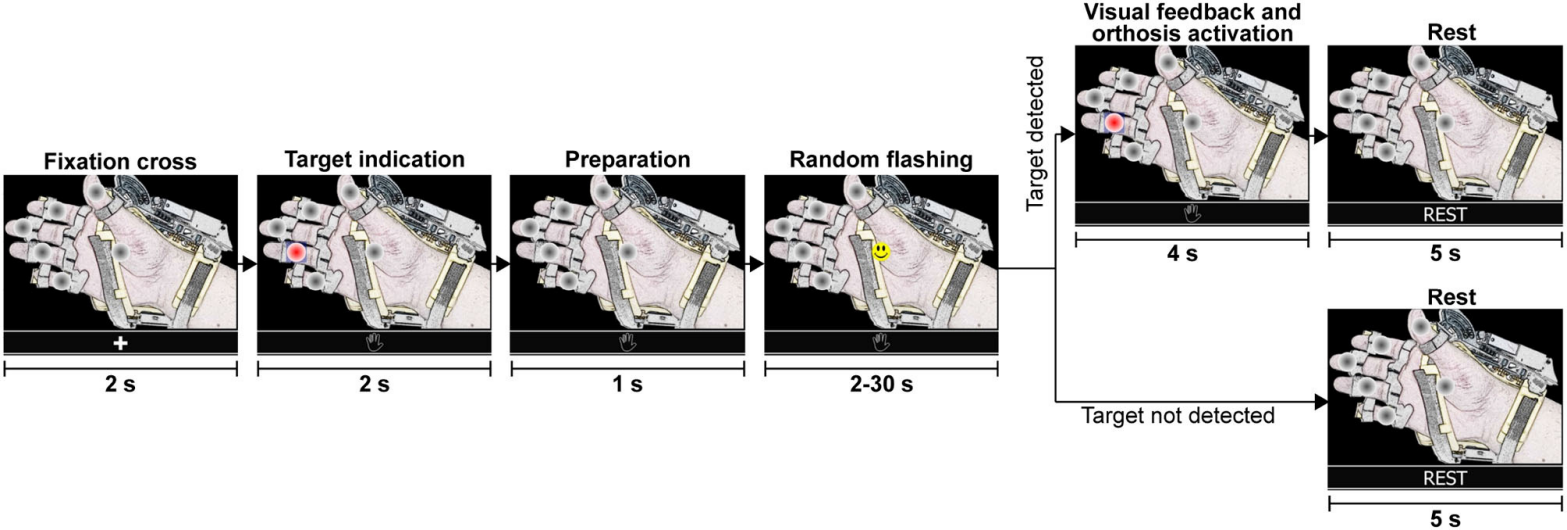
Graphical representation of a single test run. During the random flashing, the BMI tried to detect an evoked potential while the participant focused attention on the specified target option. If the system detected the correct option, the robotic device performed the selected action. If the target was not detected within 30 s during the random flashing, the system went directly to the rest period before starting a new test run with the fixation cross.

In the next stages of the experiment (online tests), subjects were indicated to focus attention on the specified target option until the BMI recognized a P300 response for one of the flashing elements. All online attempts (shown in Figure 8) are similar to the calibration runs. The interface presented a fixation cross to indicate the beginning of a test run, followed by the presentation of the target option and preparation time. Then, the random flashing started, and the BMI tried to recognize an evoked response. If the system detected in <30 s the correct option, the hand-orthosis performed the selected movement; otherwise, nothing happened. Finally, there were 5 s of resting time before starting another attempt.

Some online runs were performed with the robotic device disabled. In these cases, subjects did not wear the hand-orthosis, and the system did not send the control signals to the device. Table 1 indicates the number of online attempts performed by each participant with and without the Hand of Hope. In this way, we collected three datasets for each participant, the calibration data, the online test data without the robotic device, and the EEG recordings with the hand-orthosis.

### 2.4. Data Analysis

#### 2.4.1. ERP Analysis

The calibration data recorded in our experiments was used to analyze the ERP responses of each participant. In this study, we pre-processed and validated the training epochs of the target and non-target classes to calculate the average waveforms of both conditions. To determine the ERPs, we used the filtered signals obtained with the bandpass filter of 4–40 Hz. We considered 200 ms of pre-stimulus samples and 800 ms of post-stimulus time points.

Significant ERP peaks were identified through a statistical test of the ERP amplitude at each time point and channel with the corresponding probability density function (PDF) of the pre-stimulus interval. We estimated the PDF of the pre-stimulus segment of each channel with the non-parametric kernel density estimation method (Bowman and Azzalini, 1997). The upper and lower limits of the PDFs were then computed for a significance level of α = 0.05, i.e., significant ERP responses are those for which the probability values under the PDF of the corresponding pre-stimulus are higher than 1−α/2 or smaller than α/2.

Significant ERP responses in the target class indicate that the interface is eliciting evoked potentials when the subject perceives a flashing event of a target option. On the other hand, it is expected not to observe significant evoked potentials in the non-target class because the subject is not attending these events.

#### 2.4.2. Classification Model Evaluation

In this study, we evaluated the accuracy of the machine learning model of the BMI for each subject by applying five-fold cross-validation on the calibration data (Berrar, 2019). This method is useful to estimate the prediction error and accuracy of a model when the number of available observations is limited, and it is not possible to split the complete dataset into training data and test data. For this assessment, we report the accuracy *acc*_*i*_ of each class *i* ∈ {target, non-target} (the proportion of samples of class *i* predicted in this class correctly), and the weighted model accuracy *acc*_*w*_ = 0.5 × (*acc*_target_ + *acc*_non-target_). We used the weighted accuracy because the training data sets are highly unbalanced, and we want to avoid a bias toward the non-target class.

Additionally, the significance levels of the model accuracies were calculated with a permutation test (Good, 2006). In this methodology, the null hypothesis indicates that observations of both classes are exchangeable so that any random permutation of the class labels produces similar accuracies to the obtained with the non-permuted data. The alternative hypothesis is accepted when the model accuracy is an extreme value in the empirical distribution built with *m* random permutations. When the alternative hypothesis is accepted, we can say that the cross-validated accuracy is above the chance level.

#### 2.4.3. Online Evaluation

We assessed the online BMI performance in terms of selection accuracies, detection times and ITR. These parameters are computed through Equations (7)–(9), where *acc*_*online*_ is the online accuracy, *n*_*sel*_ is the number of correctly selected targets, *n*_*att*_ is the number of attempts to select a target or test runs, *B* is the information-rate transmitted (bits), *n*_*c*_ is the number of flashing circles, and *t*_*avg*_ is the average time from target indication to target selection (detection time in seconds).

(7) $$\begin{array}{l} acc=\frac{n_{sel}}{n_{att}}\times 100\%. \end{array}$$

(8) $$\begin{array}{l} B={\text{log}}_{2}n_{c}+\left(acc\right)\left({\text{log}}_{2}acc\right)+\left(1-acc\right){\text{log}}_{2}\frac{1-acc}{n_{c}-1}. \end{array}$$

(9) $$\begin{array}{l} ITR=60\times \frac{B}{t_{avg}}. \end{array}$$

## 3. Results

### 3.1. ERP Responses

**Figure 9:**
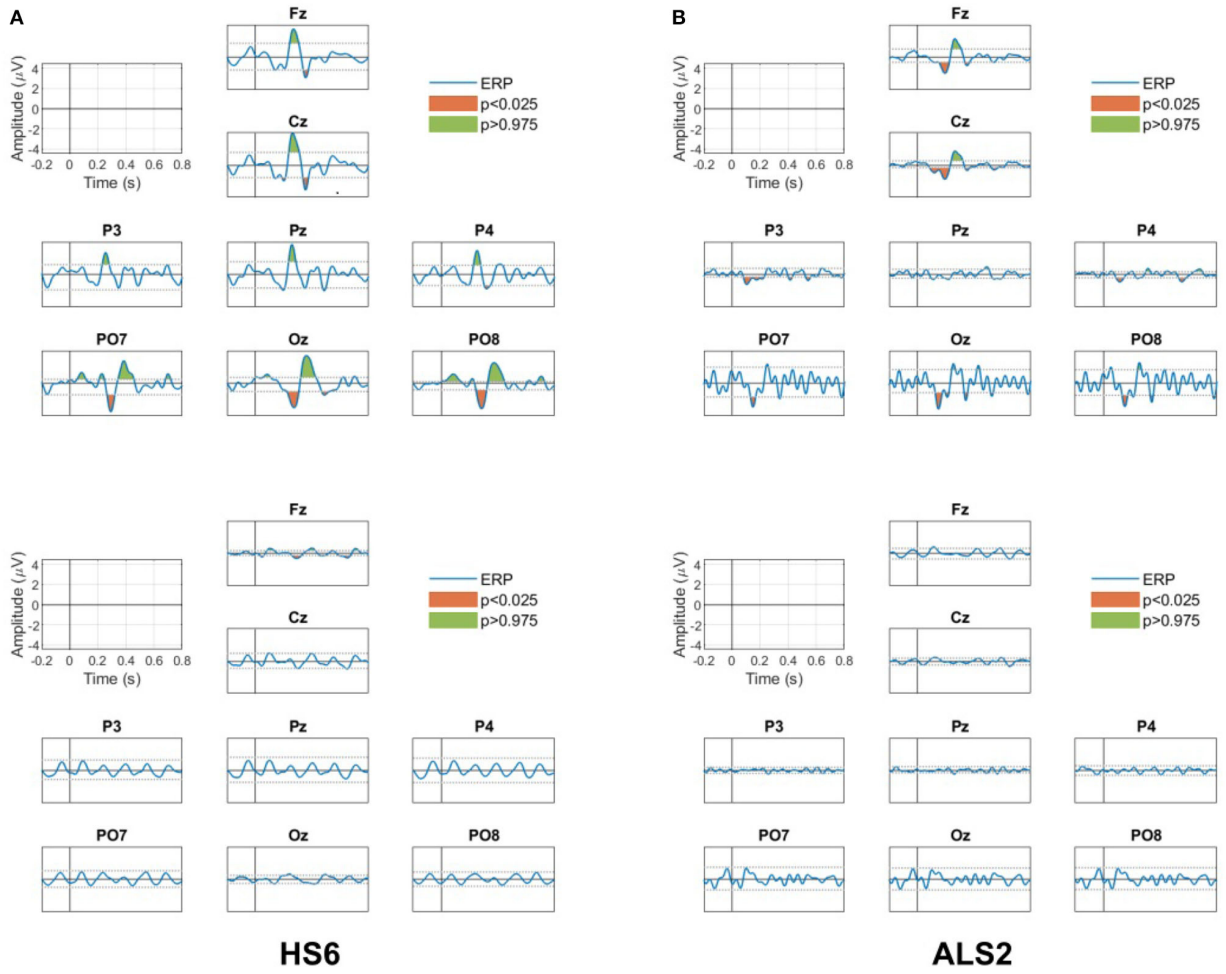
ERP responses for all channels for the target (upper panels) and non-target (bottom panels) conditions for **(A)** the healthy participant HS6 and **(B)** the patient ALS2. Green and red areas in the ERP for the target condition are the positive and negative peaks that presented significant differences (*p* < 0.05, two tail test) with the estimated PDF of the baseline period. No significant peaks are observed in the ERP for the non-target condition.

Figure 9 shows the results of the ERP analysis for one of the healthy subjects (HS6) and one of the ALS patients (ALS2). This analysis is presented for all channels separately for the target and non-target conditions. For the two participants, significant positive and negative peaks (*p* < 0.05, two-tail test) are observed in the ERP for the target condition (top figures), while no significant ERP peaks (*p* > 0.05, two-tail test) are observed in the non-target condition (bottom figures).

For the healthy subject HS2, the ERP in the target condition shows (i) a positive peak between 250 and 450 ms in all channels (the P300 response), (ii) a negative peak between 450 and 550 ms in the frontal Fz and the central Cz channels (possibly a late negativity), and (iii) an early negativity around 200 and 250 ms in the parieto-occipital (PO7 and PO8) and occipital (Oz) channels. Note that none of these features are observed in the non-target condition.

For the patient ALS2, the ERP in the target condition shows (i) the positive peak representing the P300 response between 250 and 450 ms in the frontal Fz and the central Cz channels, and (ii) an early negativity around 200 and 250 ms in the frontal and the central (Fz and Cz), the parieto-occipital (PO7 and PO8) and the occipital (Oz) channels. Note that these significant peaks are not observed in the non-target condition.

Similar observations are also present in the rest of the participants and indicate the existence of significant task-related evoked activity that is used by the proposed BMI system to recognize the stimulus the user is attending.

### 3.2. Classification Model Accuracy

Table 2 contains the classification accuracies estimated with five-fold cross-validation for each participant. The mean accuracy for the target class was 78.7%, for the non-target class was 85.7%, and the weighted accuracy was 82.2%. Only the model performance for two participants was below 70% (HS17 and ALS3), whereas three participants obtained accuracies above 90% (HS6, HS7, and HS10). The best classifier performance was 95.8%, and the worst was 66.5%. All these results are similar to those reported in other similar works (Wang and Chakraborty, 2017; Won et al., 2018).

**Table 2:** Classification accuracies (%) estimated with cross-validation for the target and non-target classes.

**Subject**	**Target**	**Non-target**	**Mean**
HS1	76.1	85.1	80.6
HS2	76.2	85.3	80.8
HS3	85.3	90.4	87.8
HS4	81.4	88.6	85.0
HS5	78.7	85.3	82.0
HS6	93.3	98.4	95.8
HS7	90.5	95.2	92.8
HS8	86.3	90.5	88.4
HS9	83.8	87.9	85.9
HS10	87.0	94.4	90.7
HS11	77.2	85.5	81.4
HS12	75.7	81.3	78.5
HS13	72.5	78.9	75.7
HS14	87.2	91.2	89.2
HS15	71.9	79.4	75.7
HS16	73.7	80.4	77.1
HS17	61.6	76.1	68.9
HS18	71.2	81.5	76.3
ALS1	80.9	87.6	84.2
ALS2	71.5	76.3	73.9
ALS3	63.2	69.8	66.5
ALS4	79.5	86.7	83.1
ALS5	73.0	83.4	78.2
ALS6	86.5	91.5	89.0
ALS7	84.5	89.9	87.2
ALS8	77.8	86.5	82.2
Mean	78.7	85.7	82.2
Std	7.8	6.5	7.1

*The fourth column indicates the model accuracy (mean value)*.

In the permutation tests, the classification accuracies for all participants were significant (*p* < 0.001, 10,000 random permutations). These results indicate that the machine learning model implemented in our BMI can discriminate between EEG epochs of the target and non-target classes. However, if we want to avoid selection errors in the online operation, it is important to consider a multi-trial strategy because the error rates are not zero. For this reason, the interface controller processes consecutive labels returned by the classification stage to determine the desired option.

Finally, we performed a Wilcoxon rank sum test and no significant differences were observed between the classification accuracies of the HS group and the ALS group (*p* = 0.60). We can say from this result that ALS participants can operate the BMI just as HS would.

### 3.3. Online Performance and Detection Times

**Figure 10:**
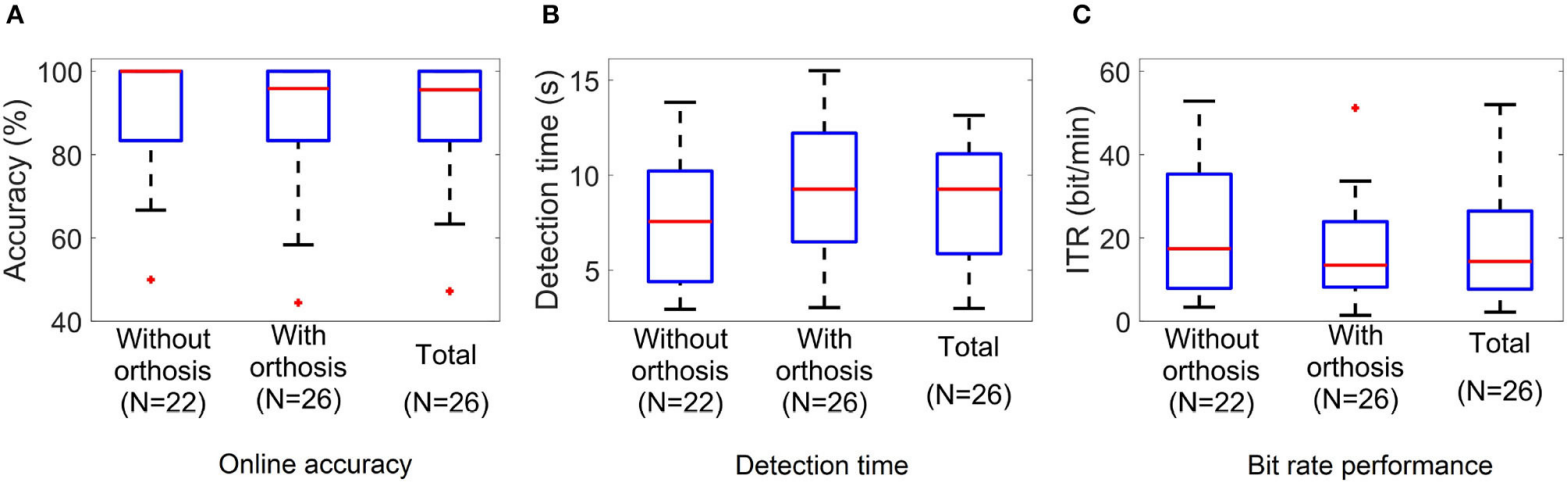
Boxplots of the **(A)** accuracy, **(B)** target detection time, and **(C)** ITR values of the BMI online test. *N* indicates the number of participants who performed the experiment under each condition.

Tables 3, 4 summarize the results obtained in the online evaluation of the proposed BMI. The distribution of the online accuracies, detection times, and ITRs are represented in Figure 10. From these results, we can observe that around 46% of the participants achieved an accuracy of 100% in the online tasks. The mean online accuracy was 89.83%, and only three participants obtained accuracies below 75% (HS16, HS17, and ALS5). We can say from this performance evaluation that the implemented BMI decodes the user's intentions effectively in most cases, and users could manipulate the hand-orthosis without much hassle in more complex tasks. However, it is essential to improve the system performance for those users who can not achieve high detection rates.

**Table 3:** Online classification performance obtained in the evaluation of the mind-controlled hand-orthosis.

	**Accuracy (%)**		
**ID**	**Without orthosis**	**With orthosis**	**Total**
HS1	75.00	83.33	80.00
HS2	100.00	88.89	94.44
HS3	100.00	100.00	100.00
HS4	100.00	100.00	100.00
HS5	100.00	100.00	100.00
HS6	100.00	100.00	100.00
HS7	100.00	100.00	100.00
HS8	100.00	100.00	100.00
HS9	100.00	91.67	96.67
HS10	100.00	100.00	100.00
HS11	88.89	100.00	94.44
HS12	94.44	88.89	91.67
HS13	83.33	83.33	83.33
HS14	100.00	100.00	100.00
HS15	72.22	77.78	75.00
HS16	66.67	58.33	63.33
HS17	50.00	44.44	47.22
HS18	88.89	66.67	83.33
ALS1	NA	83.33	83.33
ALS2	NA	75.00	75.00
ALS3	NA	100.00	100.00
ALS4	NA	100.00	100.00
ALS5	77.78	66.67	73.33
ALS6	100.00	100.00	100.00
ALS7	100.00	88.89	94.44
ALS8	100.00	100.00	100.00
Mean	90.78	88.35	89.83
Std	14.12	15.38	13.87

*Results are reported separately for each condition of orthosis usage (with or without orthosis). The fourth column (total) includes the results for all the test runs regardless of if the orthosis was used or not. NA indicates absence of validation runs under that experimental condition. The last two rows show the mean and standard deviation (std) of the accuracies*.

**Table 4:** Average detection times and ITRs obtained in the online evaluation of the proposed BMI.

**ID**	**Average selection time (s)**			**ITR (bit/min)**		
	**Without orthosis**	**With orthosis**	**Total**	**Without orthosis**	**With orthosis**	**Total**
HS1	10.22	11.26	10.87	7.01	8.25	7.72
HS2	10.67	12.47	11.52	14.53	8.78	11.18
HS3	5.43	7.62	6.74	28.56	20.37	23.01
HS4	6.35	6.72	6.54	24.43	23.08	23.73
HS5	8.68	13.56	11.12	17.86	11.44	13.95
HS6	2.94	3.03	2.98	52.83	51.20	52.00
HS7	4.18	4.61	4.40	37.08	33.63	35.27
HS8	4.33	6.49	5.41	35.82	23.91	28.67
HS9	10.56	12.21	11.18	14.69	9.72	12.32
HS10	3.10	4.93	4.02	50.01	31.43	38.60
HS11	10.13	11.05	10.62	10.80	14.04	12.13
HS12	7.61	8.50	8.04	16.92	12.87	14.75
HS13	13.41	12.34	13.14	6.93	7.52	7.07
HS14	3.57	5.94	4.76	43.45	26.10	32.61
HS15	9.54	11.19	10.40	6.84	7.00	6.89
HS16	11.28	15.50	12.84	4.75	2.47	3.67
HS17	7.51	12.61	9.91	3.39	1.45	2.19
HS18	13.84	10.40	13.15	7.91	5.15	7.06
ALS1	NA	9.80	9.80	NA	9.48	9.48
ALS2	NA	8.72	8.72	NA	8.21	8.21
ALS3	NA	7.40	7.40	NA	20.96	20.96
ALS4	NA	10.12	10.12	NA	15.33	15.33
ALS5	9.56	14.83	11.47	8.19	3.61	5.91
ALS6	4.39	5.72	5.06	35.31	27.10	30.66
ALS7	4.84	7.19	5.94	32.05	15.23	21.67
ALS8	6.16	5.56	5.86	25.17	27.90	26.46
Mean	7.65	9.22	8.54	22.02	16.39	18.13
Std	3.26	3.30	3.03	15.34	11.69	12.50

*NA indicates absence of validation runs under that experimental condition. The last two rows show the mean and standard deviation (std) of the detection times and ITRs*.

One way to increase online accuracy is to modify the detection criteria of the interface controller. The number of processed epochs and thresholds for target and non-target classes determine the balance between detection times and classification errors. For instance, if we decrease the non-target class threshold, we can reduce the number of online errors, but it is possible to see higher detection times. Our BMI can customize these parameters for each subject, but for this study, we used the same parameters for all participants.

The average detection time observed in our experiments was 8.54 s, whereas the ITR was 18.13 bit/min. The best and worst times were 2.98 and 13.15 s, and the minimum and maximum ITRs were 2.19 and 52 bit/min. Other studies have reported similar results for P300-based BMIs. If we consider that the target population of this technology is people with ALS, these response times are acceptable for many applications such as spellers and smart houses. In the case of a hand-orthosis, it is clear that it is not possible to implement an active fine control for the robotic device. However, users can select complete movements or sequences of actions using our interface. For this reason, we consider that the detection times and ITRs of our system are suitable for the movements or actions contemplated in our BMI.

Performing a Wilcoxon rank sum test to compare the HS group and the ALS group, we do not observe significant differences in any of the three performance metrics studied in this work. Online accuracies (*p* = 0.95), detection times (*p* = 0.52), and ITRs (*p* = 0.93) are similar among groups; consequently, we cannot say the BMI performance is significantly affected by the disease, at least for the disability level of the participants included in this study.

Finally, considering the 22 subjects who performed the experiment without and with orthosis, we carried out a paired *t*-test to analyze the differences in the system performance between not wearing and wearing the hand-orthosis. While no significant differences in accuracy were found between these two conditions (*t* = 1.69, *df* = 21, *p* = 0.1), the study suggests a significant impact on the detection times (*t* = −3.67, *df* = 21, *p* = 0.001) and ITRs (*t* = 3.82, *df* = 21, *p* = 0.001) produced by the use of the orthosis. These differences may be explained by induced noise mixed with the EEG when the participant wears the hand-orthosis. The linear motors and the power supply of the robotic device produce noise components that can be observed in the electroencephalogram. In this way, the system detects and rejects contaminated epochs more often when the device is turned on and in contact with the user's skin, increasing the detection time. Fortunately, the penalization in the system performance is only 1.57 s, which is not a problem in a P300-based BMI if we consider the typical reaction times of these systems.

## 4. Discussion

In this work, we presented the development and evaluation of a P300-based BMI coupled with a robotic hand-orthosis. With this system, ALS patients can manipulate each finger of a hand mentally or perform a sequence of movements of one or more fingers. Because the BMI uses the P300 paradigm, the number of possible movements is not limited, and the BMI can provide a range of options for different needs. Our system is able to perform the thumb opposition movement or movements with any combination of fingers, we can also configure the orthosis to be initially closed and perform the extension-flexion of the fingers, the initial position and angular range of the movements of the orthosis can also be controlled, this allows to adapt the system to the individual characteristics of the users (e.g., spasticity, rigidity, level of hand motor impairment), however, for this initial evaluation, we wanted to test the general performance of the interface at the most individual level (single finger movements) and with the most complex movement (all fingers simultaneously), having a total of six possible movements.

In the experiments conducted with HS and ALS patients, we observed event-related activity for the target class in the EEG recordings of all the participants. Additionally, the classification accuracies estimated with cross-validation were above the chance level for all subjects. Finally, in the online tests, both HS and ALS participants were able to control the hand-orthosis with the interface. Only three subjects obtained online accuracies below 75%, and 46% of the study subjects completed all the test runs without errors. These results indicate that our interface can discriminate successfully between target and non-target flashing events, and we can expect that most healthy people and ALS patients with mild to moderate general disability levels (according to the ALSFRS-R scale) are potential users of this assistive technology. After the tests, the users were informally asked about their experience; being the first experience of the subjects with a BMI technology, most of them showed amazement, many of them showed deeply interested and asked about the details of operation and current state of this technology. Some users reported mild eyestrain during the BMI training stage, but all reported feeling physically comfortable during the test.

In this kind of application, it is essential to achieve high accuracies to avoid the user's frustration and increase the chance of acceptance of this technology for daily life use. Although most of the participants obtained low error rates in the conducted experiments, we must find strategies to improve the system performance for users with low classification accuracies. As long as the training data contains observable event-related activity for the target class and the classification model accuracy is above the chance level, we can modify the detection criteria implemented in the interface controller to improve the online performance and adapt the interface to the user's needs. Another possibility would be the modification of the stimulus presentation and the graphical user interface. Some studies have suggested that variations in the visual stimuli characteristics produce variations in the ERPs waveforms, and thus an impact on the BMI performance (Speier et al., 2017; Li et al., 2020).

To our knowledge, this is the first report of a non-invasive P300-based system with multiple possible selections coupled with a robotic hand-orthosis that has been tested with ALS patients. Despite there are previous recent reports of P300-BMIs to control hand-orthosis or artificial hands (Stan et al., 2015; Syrov et al., 2019), these systems were tested only with healthy people, and consider applications mainly for stroke survivors. Stan et al. (2015) presented a system where a hand-orthosis is controlled through a P300-based BMI; however, the system contains only three possible selections (turn on, close, and open orthosis) that allow the flexion-extension of the five fingers simultaneously, while our system allows the passive flexion-extension of a single finger at a time. The evaluation of these fine motor movements is particularly important in ALS patients since this disease is directly associated with the degeneration of the corticospinal tract (Sarica et al., 2017), which is involved in fine digital movements (Levine et al., 2012). Syrov et al. (2019) developed a P300-BMI approach to control each finger of an wired, artificial phantom hand which does not perform the passive flexion-extension of the users fingers. In their system, the visual stimuli are shown through LEDs placed directly on the fingers of the artificial hand; this configuration, in addition to the absence of wireless communication to the robotic hand, could bring additional difficulties to test the system with ALS patients due to their motor limitations. On the other hand, Gull et al. (2018) proposed a prototype intended to be used with ALS patients that includes a BMI and a robotic glove to assist hand grasping; nevertheless, the robotic glove (Nilsson et al., 2012) covers only three fingers (thumb, middle, and ring), and the implementation of the BMI paradigm, glove control, and clinical tests were reported inconclusive.

The datasets of each participant collected in this study are publicly available with the idea of contributing to the development of new processing and classification methods for BMI systems. The inclusion of datasets of ALS participants increases the available information containing EEG recordings for BMI purposes and facilitates the improvement of BMI-based tools for patients. Furthermore, the ERPs could be used to investigate potential electrophysiological biomarkers of ALS (McCane et al., 2015; Lange et al., 2016), which would help to understanding the neurodegenerative mechanisms of the disease.

In conclusion, the results presented in this work show the capability of our mind-controlled hand-orthosis to be used with no need of adaptations for ALS patients with moderate level of disability. Future work will focus on increasing the sample size of ALS users and investigating the effect of longitudinal use of the system on patients. We will also modify the available options of the interface to test more realistic scenarios. Our system could represent the basis for developing more practical tools, such as a portable orthosis that responds to other biosignals in addition to the EEG and that is adaptable to the degree of disability of the users. Our system could also be modified to communicate with other wireless systems (e.g., smart homes).

For this initial evaluation, we tested our system's effectiveness and efficiency in terms of accuracy and ITR, respectively; for our future work, we will adopt an user-centered design (UCD) approach (Liberati et al., 2015; Schettini et al., 2015; Riccio et al., 2016; Kübler et al., 2020) and include the evaluation of satisfaction by consulting and registering the opinion of primary (ALS patients) and secondary (caregivers) end-users through formal interviews. Feedback from patients and their caregivers will help to develop a more customizable system according to the individual characteristics and needs of each user. The UCD approach will also help us to properly identify and correct the present limitations in order to improve the usability of our system in daily life.

## Data Availability Statement

The datasets generated and analyzed for this study are available upon request to the corresponding author.

## Ethics Statement

All participants volunteered for the study and provided informed consent before the experimental sessions.

## Author Contributions

JD, OM-M, JG, RC, HM, and JMA participated in the study design, experiments, and manuscript writing. JD and OM-M designed and implemented the brain-machine interface. JD, OM-M, and JA performed the acquisition and analysis of data. JD recruited the healthy participants. RC and HM selected the amyotrophic lateral sclerosis patients.

## Conflict of Interest

The authors declare that the research was conducted in the absence of any commercial or financial relationships that could be construed as a potential conflict of interest.
